# Parke, Davis & Co.

**Published:** 1888-02

**Authors:** 


PARKE, DAVIS & Co., the enterprising and progressive drug house of Detroit, and doing business also in New York, sends a New Year's greeting to the editors of the journal. We appreciate the friendly token and cordially reciprocate the same. Their card is gotten out with elegant taste. See their interesting Talk to Physicians on the fourth cover page of this journal.

We are pleased also to acknowledge the receipt of a splendid portrait of Sir Morill McKenzie from this excellent house. Sir Morill McKenzie is the eminent physician of London, who is supposed to have given a correct diagnosis and operated for the throat trouble of the Crown Prince of Germany, although opposed in his views by the highest German authority.

